# MINIMALLY INVASIVE SURGERY FOR PSEUDOPAPILLARY NEOPLASM OF THE PANCREAS

**DOI:** 10.1590/0102-6720201600020008

**Published:** 2016

**Authors:** Guilherme Naccache NAMUR, Thiago Costa RIBEIRO, Marcelo M. SOUTO, Estela Regina Ramos FIGUEIRA, Telesforo BACCHELLA, Ricardo JUREIDINI

**Affiliations:** Hospital de Clínicas, Faculty of Medicine, University of São Paulo, São Paulo, SP, Brazil

**Keywords:** Pancreatic neoplasms, Minimally invasive surgical procedures, Pancreatectomy, Laparoscopy, Pancreatoduodenectomy

## Abstract

**Background:**

Solid pseudopapillary pancreatic neoplasia is usually a large well-circumscribed pancreatic mass, with cystic and solid areas more frequently found in young women. It is a benign pancreatic neoplasia in most cases, therefore minimally invasive surgery could be an interesting approach.

**Aim:**

Evaluate the results of minimally invasive surgery for this neoplasia.

**Methods:**

Patients with this tumor who underwent minimally invasive pancreatectomies between 2009 and 2015 in a single institution, were analyzed regarding demographic, clinical-pathological futures, post-operative morbidity and disease-free survival.

**Results:**

All were women, and their median age was 39 (18-54) years. Two patients with tumor in the head of the pancreas underwent laparoscopic pancreaticoduodenectomy, and another one underwent laparoscopic enucleation. Two patients with tumor in the neck underwent central pancreatectomy. Distal pancreatectomies were performed in the other five, one with splenic preservation. None required blood transfusion or conversion to open surgery. Two (20%) developed clinical relevant pancreatic fistulas, requiring readmission. Median length of postoperative hospital stay was five days (2-8). All resection margins were negative. Patients were followed for a median of 38 months (14-71), and there was no recurrence.

**Conclusions:**

Minimally invasive surgery for solid pseudopapillary pancreatic neoplasia is feasible for tumors in different locations in the pancreas. It was associated with acceptable morbidity and respected the oncologic principles for treatment.

## RESUMO

**Racional:**

Tumores sólidos pseudopapilíferos do pâncreas são em geral massas circunscritas, com componentes sólidos e císticos, mais frequentemente encontrados em mulheres jovens. Na maioria dos casos, são neoplasias benignas e portanto, a abordagem minimamente invasiva pode ser considerada vantajosa.

**Objetivo:**

Avaliar os resultados das pancreatectomias laparoscópicas em pacientes com esses tumores.

**Métodos:**

Foram avaliados pacientes submetidos à pancreatectomias minimamente invasivas em um única instituição. Foram analisadas variáveis demográficas, clinicopatológicas, complicações pós-operatórias e sobrevida.

**Resultados:**

Todos os pacientes eram mulheres e a idade mediana foi de 39 (18-54) anos. Duas pacientes com tumores na cabeça do pâncreas foram submetidas à duodenopancreatectomia e outra à enucleação. Duas pacientes com tumores no colo do pâncreas realizaram pancreatectomia central. Outras cinco com neoplasias no corpo e cauda foram submetidas à pancreatectomia distal, uma delas com preservação do baço. Nenhuma necessitou de conversão para laparotomia. Houve duas fístulas pancreáticas clinicamente relevantes (20%). O tempo mediano de internação foi de cinco (2 a 8) dias e duas pacientes foram reinternadas (20%). Todas as margens de ressecção foram negativas e após seguimento mediano de 38 (14-71) semanas, todas estavam livres de doença.

**Conclusão:**

As ressecções pancreáticas minimamente invasivas de tumores sólidos pseudopapilíferos são factíveis para tumores em diferentes localizações no pâncreas, com morbidade aceitável obedecendo-se os princípios oncológicos para o tratamento dessas neoplasias.

## INTRODUCTION

Solid pseudopapillary neoplasm (SPN) is a rare pancreatic neoplasia with low malignancy potential that occurs mostly in women (87.8%), between 20-40years with a mean age of 28 years^13^ ^,^ ^17^ ^,^ ^22^ ^,^ ^30^. 

Usually asymptomatic, it can cause vague abdominal pain, abdominal mass, nausea, vomiting or weight loss^10^ ^,^ ^13^. Although it is an indolent disease^10^ ^,^ ^28^, local invasion or distant metastasis are found in 9-15% at diagnosis^10^ ^,^ ^13^ ^,^ ^28^.

Most SPN are discovered incidentally after cross section images exams such as computed tomography (CT) or magnetic resonance (MRI), which usually are sufficient for the diagnosis. Tumor markers are generally normal. Usually they are located in body and tail of the pancreas (59.3%), but also can be found in head and uncinate process in 36% of cases^13^ ^,^ ^30^.

Free margins resection is the only curative treatment for them. Nodal involvement is almost anecdotal^27^; therefore, lymphadenectomy should be avoided. As most patients are young and shall be cured after resection, concerns about endocrine and exocrine insufficiency are important, because they can impair quality of life^21^ ^,^ ^24^. Another issue that may have an impact in long-term results is the large incisions required in open approach which are associated with incisional hernias and poor aesthetic outcome. Under this context, minimally invasive procedures seems as an ideal approach to patients with this disease, as it has been shown that it is related to less blood loss, shorter hospital stay and better cosmesis^18^.

Gagner described the first laparoscopic distal pancreatectomy in 1994^6^, but it was not until the second half of the years 2000 that the procedure became usual in clinical practice^14^ ^,^ ^18^ ^,^ ^19^. Others pancreatic resections, such as enucleations, central pancreatectomies or pancreatoduodenectomies are even more rarely reported in literature^2^ ^,^ ^8^ ^,^ ^26^. The aim of the present study was to analyze the results of a series of patients with SPN operated by minimally invasive approach in a single institution.

## METHODS

Were analyzed retrospectively from a prospective maintained database, patients with radiologic and pathologic diagnosis of SPN who underwent laparoscopic pancreatic resection from November 2009 to March 2015 in Hospital das Clínicas, Faculty of Medicine, University of São Paulo, São Paulo, Brazil. Patients' demographic features, such as sex, age, BMI, American Society of Anesthesiology classification (ASA), and clinical pathologic features, such as tumor size and location were reviewed. Intra-operative parameters like type of resection, operative time, blood loss were assessed. Post-operative complications were defined according to the consensus of the International Study Group for the Pancreas (Pancreatic Fistula, Delayed Gastric Empting)^1^ ^,^ ^29^ and their severity graduated according to the Clavien-Dindo classification^5^. Long-term outcome was measured as disease free survival in months and also the presence of new onset impaired exocrine or endocrine insufficiency. When necessary, numbers were presented as medians.

### Surgical technique

**FIGURE 1:**
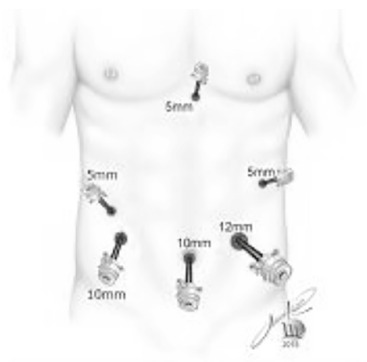
Trocars disposition

All patients were operated under general anesthesia in a reverse Trendelemburg position with legs splitted apart and the surgeon between patients' legs. Trocars were disposed according to Figure 1.

**FIGURE 2:**
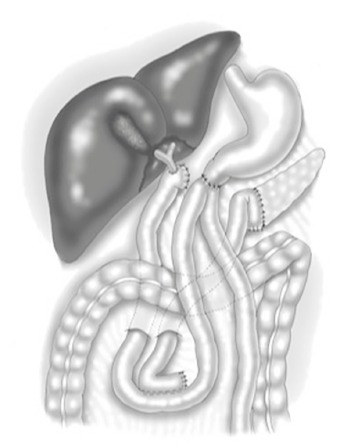
Pancreatoduodenectomy with pylorus preserving technique

Pancreatoduodenectomy was performed with pylorus preserving technique. Reconstruction was done with a separated loop for the pancreatojejunostomy (Figure 2) as previously described^16^.

In central pancreatectomy, proximal pancreas was transected with a laparoscopic stapler and the distal pancreas was drained by a Roux-en-Y pancreatojejunostomy. To distal pancreatectomy, the pancreatic stump was closed either with a laparoscopic stapler or hand sewing. 

## RESULTS

From November 2009 to March 2015, 10 patients were operated by minimally invasive approach for SPN in the Hospital das Clínicas, Faculty of Medicine, University of São Paulo, São Paulo, Brazil. Demographic and pathologic data are summarized in Table 1. All patients were women, and the median age was 39 years (18-54). Only one patient (patient 7) presented with lumbar pain, all others were asymptomatic and had an incidental diagnosis. The median BMI was 25 kg/m² (19-42.3), and the American Society of Anesthesiologists classification was III in only one. Tumors were localized in pancreatic tail or body in five patients; in the neck in two, and in three in the head. Mean tumor size was 3.6 cm (1.3-8 cm). One patient had a synchronic neuroendocrine tumor in the pancreatic tail, which was biopsied by endoscopic ultrasonography (EUS). 

**TABLE 1:** Patients demographics and clinical-pathological data

Patient	Age (yrs)	BMI (kg/m^2)^	ASA	Location	Tumor size (cm)
1	37	20,8	I	Body	3,7
2	29	25	I	Head	8
3	45	37	II	Body	3,5
4	41	28	II	Head/neck	1,3
5	18	22	I	Head	4,5
6	35	20,3	I	Head	2,5
7	54	24,1	II	Body/tail	4,5
8	49	27	I	Body and tail*	3,2/ 1,1*
9	53	42,3	III	Tail	4,5
10	29	21	II	Head/neck	2,3

* Syncrhornic neuroendocrine tumor

Different procedures were performed according to tumor location. Surgical outcome and long term follow up is summarized in Table 2.

**TABLE 2:** Short and long term outcome

Patient	Procedure	Operative time (min)	Nodal status	Pancreatic fistula	Re-operation	LOS (days)	Clavien	Death	Recurrence	DFS (months)
1	DPS	135	0/1	No	No	4	0	No	No	71
2	PD	800	0/9	B	No	8 (9)	2	No	No	63
3	SPDP	140	0/1	C	Yes	2	3B	No	No	64
4	CP	250	0/8	A	No	8	1	No	No	52
5	Enucleation	120	0/0	A	No	4	1	No	No	38
6	PD	375	0/8	No	No	8 (7)	0	No	No	38
7	DPS	210	0/2	No	No	5	0	No	No	37
8	DPS	200	0/13	A	No	6	1	No	No	23
9	DPS	240	0/6	A	No	4	1	No	No	22
10	CP	150	0/0	A	No	5	1	No	No	14

CP= central pancreatectomy; DPS=distal pancreatectomy with splenectomy; PD=pancreatoduodenectomy; SPDP=spleen preserving distal pancreatectomy; LOS=lenght of stay; DFS=disease free survival

**FIGURE 3:**
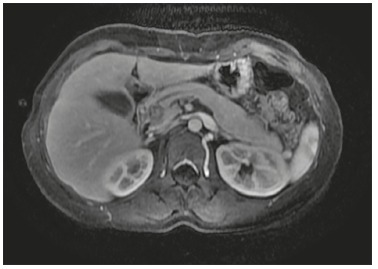
Tumor in the head of the pancreas (patient 6)

Head of the pancreas (Figure 3)

Two patients (2 and 6) with tumor in the head of the pancreas underwent laparoscopic pancreaticoduodenectomy with double loop reconstruction. Operative time was 800 min for the first patient and 345 min for the second, there was no important intra-operative bleeding and they were discharged from hospital in 9^th^ and 7^th^ postoperative day, respectively. Both had soft pancreatic parenchyma with small main pancreatic duct (<3 mm) and no biliary dilatation.

Patient 2 had a grade B fistula (ISGPF) and biliary fistula (Clavien 2B) and was readmitted in 17^th^ postoperative day due to fever, and responded to antibiotics. At follow-up, she developed hepaticojejunostomy stricture requiring surgical revision after 10 months, and a new anastomosis was performed. 

A third patient with a tumor in the head of the pancreas (patient 5) had a lesion in the anterior surface of the pancreas, distant from the main pancreatic duct and biliary duct. Was performed a pancreatic enucleation and she developed a transient pancreatic fistula (type A, Clavien 1) and was discharged on 4^th^ postoperative day. 

### Pancreatic neck

Two patients with tumor in the pancreatic neck (patients 4 and 10) underwent central pancreatectomy with Roux-en-Y pancreaticojejunostomy reconstruction. Operative time was 250 and 150 min without any significant intra-operative bleeding. Both patients developed grade A pancreatic fistula (Clavien 1) with no other complications. 

**FIGURE 4:**
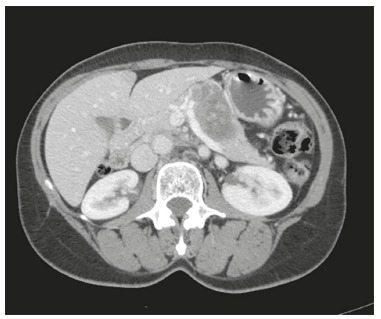
Tumor in the pancreatic body an tail (patient 7)

Pancreatic body and tail (Figure 4)

Five patients (1, 3, 7, 8 and 9) underwent distal pancreatectomy and splenic preservation was achieved in one (number 1). The median operative time was 200 min (135-240). None required blood transfusion. There were two grade A pancreatic fistula (Clavien 1) and one grade C (Clavien 3B, patient 1) who developed an organized retro-gastric collection requiring reoperation.

None of the ten cases presented delayed gastric emptying, postoperative bleeding or other complications. Morbidity rate was 70%, but there was only one severe complication (Clavien>3). All resection margins were negative, and there was no lymph node involvement. Median length of postoperative hospital stay was five days (2-8) and two patients required readmission (patients 2 and 3). They were followed for a median of 38 months (14-71), and there was no recurrence, signs of exocrine insufficiency or new onset diabetes.

## DISCUSSION

SPN occurs mostly in young females and only 12.2% of these tumors are diagnosed in men. Symptoms are nonspecific, and diagnosis is often incidental, after cross section imaging exams^28^, what can explain the increasing number of reported cases after years 2000^13, 22^.

Laboratory exams and tumor markers are non specific. On image, SPN presents as an encapsulated hypodense mass, with solid and cystic components, usually with peripheral capsule and occasional calcification without ductal dilatation or atrophy of pancreatic parenchyma and occasionally contrast enhancement within the mass. MRI is slightly superior in identifying hemorrhage, cystic degeneration and the presence of capsule. The tumor can displace adjacent organs^3^. Percutaneous biopsy provides diagnosis in 61% of the cases, and by EUS in 69.5%^13^ ^,^ ^22^, however, there is a risk of tumor dissemination and also procedures related complications, therefore in most case CT or MRI are sufficient for the correct diagnosis^12^. Differential diagnosis includes adenocarcinoma, mucinous cystic neoplasm, pancreatic pseudocyst, serous cystic neoplasia and IPMN^12^ ^,^ ^22^. 

SPN are classified as a low-grade malignant neoplasm by the WHO. Criteria which distinguish the solid pseudopapillary carcinoma, by the 2010 WHO classification, are angioinvasion, perineural invasion and deep invasion of the surrounding pancreatic parenchyma. Some studies suggested that some histological features, such as extensive necrosis, nuclear atypia or pleomorphism, dedifferentiation, DNA aneuploidy, high mitotic rate, immunohistochemistry findings of expression of Ki-67 and sarcomatoid areas are associated with aggressive behavior^10^ ^,^ ^28^, nevertheless the disease's behavior can be unpredictable^22^. Tumor size ≥8 cm, microscopic malignant features, and stage IV condition (systemic metastasis and peritoneal seeding), are predictive factors for recurrence^10^. Nodal involvement is extremely rare occurring only in 1.6% of cases and distant metastasis in 7.7-15%^13^ ^,^ ^22^, usually in the liver or peritoneum and omentum^28^, and the average time to relapse is four years; however, the small number of series published makes this analyses difficult. Surgery is the only curative treatment and is indicated even if a R1 ressection is achieved^7^ ^,^ ^10^. 

Since it is a low-grade malignant tumor, usually occurring in young and healthy patients with a long life expectancy, parenchyma-preserving procedures are advisable, specially to avoid exocrine and endocrine insuficiency^13^. In two recent meta-analyses, pancreatic enucleations and central pancreatectomies were compared to standard resections and they concluded that both procedures have comparable short term outcomes regarding morbidity and mortality^8^ ^,^ ^9^.

Enucleation is an excellent alternative to avoid extensive procedures, such as pancreaticoduodenectomy, especially for lesions in the pancreatic head. When considering enucleation, the size of the tumor and distance to the main pancreatic duct should be carefully assessed. The patient who underwent enucleation of a lesion in the head of the pancreas was an 18 years old female, with a 4.5 cm tumor. She had a quick recovery, and was discharged in the 4^th^ postoperative day. Although simpler than others standard resections, like distal pancreatectomies, there are few reports in literature about laparoscopic pancreatic enucleations, mainly because most tumors aren't suitable for these procedures either because malignant or too close to the main pancreatic duct. The largest series in literature that compared open and laparoscopic enucleation concluded that minimally invasive procedures have similar morbidity rate with shorter postoperative hospital stay^25^.

When the tumor is located on pancreatic neck, without vascular invasion, a central pancreatectomy should be considered^3^. Even small lesions in this topography usually are close to the main pancreatic duct, what is a contraindication for enucleation. Although possibly associated with higher perioperative morbidity (up to 58%), central pancreatectomy offers excellent long-term results concerning endocrine (14%) and exocrine (7-22%) insufficiency^9^. Most reports of laparoscopic central pancreatectomy are small series of cases with no more than 10 patients^11^ ^,^ ^26^ and apparently it is as safe as open approach; however, it is not possible to draw any conclusions yet. 

Laparoscopic pancreaticoduodenectomy is a demanding and very complex procedure with challenging reconstruction, especially in patients with SPN, because they have soft pancreas, non-dilated pancreatic duct, and also an hepatic duct with small caliber. One of our two patients experienced a biliary leakage, with posterior stricture, requiring surgical revision while the second one showed no complications. In a recent meta-analyses, Lei et. al. demonstrated that minimally invasive approach is safe and is related to less operative bleeding and short hospital stay^15^; it must be considered that those were highly selected cases. The laparoscopic approach should be avoided in large tumors, especially when there is abutment in superior mesenteric/portal vein, because, although vascular invasion is rare, the vein can be easily disrupted during dissection. 

Laparoscopic distal pancreatectomy is already considered the gold-standard treatment for left pancreatic lesions; its advantages are reduced blood loss, early oral intake, shorter hospital stay, lesser use of analgesics, earlier return to activities and reduction in complications rate such as hernia and wound infection^18^ ^,^ ^20^ Since SPN occurs more frequently in young women, the esthetic result is also very important. The long-term results are similar, but oncological principles must be kept and the rupture of the specimen should be avoided, which could lead to the spread of tumor cells and recurrence.

The spleen preservation carries similar operative morbidity and decreases the incidence of perigastric varices^4^ ^,^ ^23^. In this series, the splenic preservation rate was low. Splenectomy may be required either to achieve oncologic resection or due to difficult dissection of the splenic vessels, the tumor in itself or adjacent inflammation.

In a systematic review in 2014, Law et. al were able to find only 39 SPN resected by minimally invasive approach reported in literature^13^. A multicentric study in Korea in that same year described 52 cases^10^. Most underwent distal pancreatectomies. Both studies describe excellent long-term outcome, as in this series, with no recurrence at all. To our knowledge, this series is the only one in literature to describe the role spectrum of pancreatic resections by minimally invasive approach for the treatment of SPN. 

## CONCLUSION

SPN is rare pancreatic lesion; however, the incidence has increased following the widespread use of imaging exams. It is a low-grade malignant tumor, with long expected survival, and frequent in young women. Surgery is the only curative treatment and minimally invasive, parenchyma-preserving procedures, are the best options of treatment.
